# Q Fever and the US Military

**DOI:** 10.3201/eid1108.050314

**Published:** 2005-08

**Authors:** Alicia D. Anderson, Bonnie Smoak, Eric Shuping, Christopher Ockenhouse, Bruno Petruccelli

**Affiliations:** * Walter Reed Army Institute of Research, Silver Spring, Maryland, USA;; † Ireland Army Community Hospital, Fort Knox, Kentucky, USA;; ‡ US Army Center for Health Promotion and Preventive Medicine, Aberdeen Proving Ground, Maryland, USA

**Keywords:** Q fever, epidemiology, pneumonia, Coxiella burnetii, military

**To the Editor:** Q fever is a zoonotic disease caused by the rickettsialike organism *Coxiella burnetii*. The disease has a worldwide distribution and can infect many different species, although cattle, sheep, and goats are the primary reservoirs (1). Transmission to humans usually occurs by inhaling dust or aerosols from infected animals, and approximately half of infected persons manifest clinical symptoms. In acute Q fever infection, the 3 main sets of symptoms are flulike syndrome, pneumonia, and hepatitis (2,3).

Q fever has military relevance not only in its potential use as a bioterrorism agent, but also because of the risk for natural infection in deployed military personnel. Thousands of cases of Q fever have been seen in military personnel since the disease was first reported in the 1930s (4). Since the most common mode of transmission is airborne, personnel do not need to have direct contact with infected animals to be exposed.

*C. burnetii* was first recognized as an infectious disease threat to US military troops serving in Iraq in 2003 during a pneumonia outbreak investigation. Nineteen cases of severe pneumonia, including 2 deaths, occurred from March 1 to August 20 (5). A case was defined as occurring in a patient with bilateral alveolar infiltrates that required intubation and mechanical ventilation. This investigation involved extensive serologic testing for possible infectious causes of pneumonia, including *C. burnetii*. Of 19 patients with severe pneumonia tested for *C. burnetii*, 3 had positive antibody titers by immunofluorescence assay (IFA). No other infectious cause was confirmed for the remaining cases of pneumonia. Although *C. burnetii* was not determined to be the cause of the pneumonia outbreak, the finding of 3 patients with positive antibody titers launched an effort to ascertain other cases of Q fever among military personnel who served in Iraq during that time.

Approximately 62 cases of pneumonia, both severe and nonsevere, occurred in Iraq from March 1 to August 20, 2003. A pneumonia case was defined as occurring in a patient with a chest radiograph suggesting pneumonia and ≥1 of the following symptoms: fever, cough, or shortness of breath. The Defense Medical Surveillance System (DMSS) was queried to determine how many patients had both predeployment and postdeployment serum samples available for Q fever testing. The Army Medical Surveillance Activity, which operates DMSS, also maintains the Department of Defense Serum Repository and stores serum from service members after mandatory HIV testing and deployment processing (6). Predeployment sera must be collected within the year before deployment.

Twenty-two soldiers had predeployment and postdeployment sera available; samples were tested for phase I and phase II antibody to Q fever by using IFA. Results showed 5 additional soldiers in whom pneumonia was diagnosed while serving in Iraq and who seroconverted to *C. burnetii* before postdeployment serum draws (Table). All predeployment antibody titers for both immunoglobulin (Ig) G and IgM were negative in these 5 soldiers, with an IFA titer of 1:16 as a cutoff.

**Table:** Postdeployment serum antibody titers to phase II antigen for Q fever in 8 US military personnel who served in Iraq, March 1–August 20, 2003*

Patient	IgG	IgM
1	1:1,024	Negative
2	1:128	Negative
3	>1:1,024	1:512
4	1:256	1:256
5	1:512	>1:1,024
6	1:512	1:512
7	1:64	1:64
8	>1:1,024	>1:1,024

*All predeployment titers were negative for immunoglobulin (Ig) G and IgM.

The initial 3 Q fever patients ascertained through the pneumonia outbreak investigation were extensively interviewed for possible exposures. All 3 patients first experienced symptoms while in northern Iraq and reported contact with domestic animals, including dogs, cats, sheep, goats, and camels. Two of the patients reported tick bites within 30 days before becoming ill, and 1 reported drinking raw sheep's milk. The 5 other patients who became ill with pneumonia also first sought care while in northern Iraq. Predeployment sera from these 3 patients were also tested for *C. burnetii* by IFA, and all samples were negative for both IgG and IgM.

Extremely limited information is available on Q fever disease prevalence in Iraq, either in animals or humans. Iraq is primarily an agricultural country, and nomadic herding takes place countrywide, except in the northernmost regions and along the eastern border, where adequate land is available for grazing livestock. The most common livestock in Iraq are cattle, sheep, and goats (7). Although herds of infected animals may exist in any region of Iraq, larger concentrations of livestock may exist in northern areas, where land is suitable for ruminants to graze. This concentration could lead to a higher risk for transmission to humans because the chance of contact with infected animals would be greater.

These data indicate the potential importance of *C. burnetii* as an infectious disease threat to US military troops in Iraq. Healthcare providers should include Q fever in their differential diagnosis of community-acquired pneumonia and consider adding doxycycline to a combined antimicrobial drug regimen to presumptively treat severe pneumonia. Future studies to be completed include case ascertainment to locate US troops who were infected with Q fever while in Iraq and in whom pneumonia or other clinical manifestations of illness may have developed.

Research was conducted in compliance with the Animal Welfare Act and other federal statutes and regulations relating to animals and experiments involving animals and adheres to principles stated in the Guide for the Care and Use of Laboratory Animals, NRC Publication, 1996 edition.
